# Predicting mental health trajectories after potentially traumatic events: a machine learning approach

**DOI:** 10.1007/s00787-026-03022-6

**Published:** 2026-04-01

**Authors:** Dunja Tutus, Tanmay Nayyar, Jörg M. Fegert, Ann-Christin Haag

**Affiliations:** 1 Clinic of Child and Adolescent Psychiatry, Psychosomatics and Psychotherapy, Ulm University, Steinhoevelstr. 3, 89075 Ulm, Germany; 2 German Center for Mental Health (DZPG), Mannheim/Heidelberg/Ulm, Germany

**Keywords:** Adolescent Brain Cognitive Development Study, Machine learning, Mental health trajectories, Potentially traumatic events, Psychosocial factors

## Abstract

This study aimed to investigate the trajectories of internalizing and externalizing problems following childhood potentially traumatic events (PTEs) and analyse a comprehensive set of baseline variables (PTEs, individual, environmental) to elucidate their predictive role as contributors to different mental health trajectories. The sample consisted of 4,141 participants (*M* = 9.48, *SD* = 0.51 years at baseline; 48.7% girls; 72.1% White) from the *Adolescent Brain Cognitive Development* study who had experienced at least one PTE. Participants’ mental health problems were assessed using the *Brief Problem Monitor* self-report form. *Latent Growth Mixture Modelling* was used to identify trajectories of youth´s internalizing and externalizing problems across the six assessments. Machine learning was utilized to investigate 37 predictors of different trajectories. Three distinct trajectories were identified for both internalizing and externalizing problems: “Resilient”, “Mild” and “Moderate chronic”. Predictors of the “Moderate chronic” versus “Resilient” trajectories were identified using machine learning. The three most important predictors of the internalizing problems trajectory were: behavioural inhibition, caregiver internalizing problems, and female gender, whereas predictors of the externalizing problems trajectory were family conflicts, screentime, and a low level of prosocial behaviour. The findings can help characterize individual variation in mental health trajectories following childhood PTEs and provide potential targets for intervention to foster mental health.

**Supplementary Information:**

The online version contains supplementary material available at 10.1007/s00787-026-03022-6.

## Introduction

Worldwide, approximately half of children/adolescents are exposed to potentially traumatic events (PTEs, e.g., interpersonal violence or abuse, war, terrorism, natural disasters, traffic accidents) [1–3]. Of these, 15.9% develop *Posttraumatic Stress Disorder* (PTSD) [4]. While the majority of individuals show resilience in adjusting to PTEs, maintaining a stable trajectory of healthy functioning, others experience mild disruptions or even severe and persistent mental health issues [5]. Research on symptom severity and change over time has identified four main trajectories: resilience, recovery (prolonged but ultimately decreasing disruption in functioning), delayed onset (disruptions that emerge after a significant delay), and chronic (continuing disruption), in both adult and child/adolescent samples [6, 7].

Numerous protective and risk factors associated with trauma adjustment have been identified. For instance, poly-victimization and trauma type (e.g., interpersonal vs. non-interpersonal; threat vs. deprivation), negative coping strategies, low self-control, family psychiatric disorders and poor functioning, low social support, racial/ethnic minority status, low socioeconomic status and female gender have all been linked to poorer trauma recovery [2, 4, 7–11]. While family, community, and school connectedness and support, peer support, higher parental education, and physical activity have been identified as robust protective factors [7, 11–15]. Many parents experience mental health difficulties following their child´s exposure to PTEs, which can contribute to the development and persistence of the child´s trauma-related problems [16, 17]. PTEs are associated with excessive recreational screentime and exposed youth are more likely to experience internet-initiated victimizations, such as cyberbullying, highlighting the need to include screentime in models for risk estimation [7, 18, 19].

Unresolved childhood trauma has been linked to academic problems, social withdrawal, delinquency, poor socioeconomic outcomes, a range of medical (e.g., cardiovascular and metabolic disorders, accelerated aging), and mental health adversities (including a wide range of internalizing and externalizing problems, as well as diagnoses like PTSD, depression, anxiety disorders, attention deficit hyperactivity disorder (ADHD), and disruptive behaviour disorders), potentially leading to lifelong impairments [12, 20–22]. However, many individuals with trauma-related sequelae are often not identified or treated promptly. A history of trauma is frequently discovered late in the treatment of other conditions [20]. To enhance healthcare and mitigate the long-term consequences of trauma, it is crucial to develop scalable prediction models based on a broad array of resilience and risk factors. These models, in addition to traditional screening methods, can help identify youth at higher risk who could benefit from ongoing symptom monitoring or timely initiation of appropriate psychological/psychotherapeutic/psychiatric interventions tailored to their individual needs. Predicting and intervening early can help reduce the delayed and enduring effects of trauma exposure, as targeted prevention is more cost-effective than treatment [23].

Most previous research has focused on only a few risk and protective factors, mainly due to limitations in sample size. Innovative approaches that utilize methods like artificial intelligence and machine learning (ML) have the capability to analyse large datasets and offer new insights to tackle longstanding research questions that were previously not adequately addressed due to small sample sizes and computational limitations [5, 24–27]. Recent studies using a ML approach have shown promising results in predicting mental health problems among children and adolescents. The majority of these studies have utilized either a cross-sectional design or electronic health records [28, 29].

To overcome these limitations, we analysed data from the *Adolescent Brain Cognitive Development* (ABCD) study, the largest long-term developmental study of child health in the United States (U.S.) [30]. Our first goal was to identify different trajectories of internalizing and externalizing problems among ABCD study participants who had experienced PTE(s). Drawing upon previous literature [6], we investigated the emergence of various trajectories—such as chronic, emerging, recovering, and resilient—within our sample. Our analysis focused on identifying whether resilient patterns would emerge as a primary feature of the observed data. The second objective was to examine a comprehensive set of risk and protective factors to distinguish between participants following chronic and resilient trajectories. We expected that predictors indicating more severe PTEs and heightened psychosocial problems would increase the likelihood of classification in chronic trajectories compared to resilient trajectories.

## Method

### Participants

The data from the ABCD study were utilized [30]. Starting in 2016, children aged 9–10, selected through probability sampling in U.S. schools, have been followed for a period of 10 years. Children were excluded if they had magnetic resonance imaging contraindications, lacked English proficiency, had severe sensory/neurological/medical/intellectual limitations, or were unwilling to complete assessments. All parents provided written informed consent and children provided assent. Institutional review board approval was obtained for each site before data collection, with central institutional review board approval granted by the University of California, San Diego [31].

Psychosocial data in the ABCD study are gathered through surveys, semi-structured interviews, and ecologically-based passive data collection methods. This information is collected from participating youth, their parents/caregivers, and teachers. We utilized data from the 5.1 release, which includes data collected between 2016 and 2022 from 21 data acquisition sites in the U.S [32]. Release 5.1 contains complete data from the baseline (*N* = 11,868), 6-month (*N* = 11,389), 1-year (*N* = 11,220), 18-month (*N* = 11,083), 2-year (*N* = 10,973), 30-month (*N* = 10,228), and 3-year follow-up visits (*N* = 10,336). Release 5.1 only includes data from approximately half of the cohort that took part in the 4-year (*N* = 4,754) and 42-month (*N* = 8,449) follow-up visits [30]. For this study, data from the baseline to the 3-year follow-up were utilized. A detailed description of the sample in the ABCD study is provided in Supplementary Table 1. Specifically, we focused on participants who had experienced at least one PTE, as reported at baseline using the *Kiddie Schedule for Affective Disorders and Schizophrenia Present and Lifetime Version* (K-SADS-PL) for DSM-5 [33]. This resulted in a final sample size of 4,141 youth (48.7% female) with an average age of 9.48 years (*SD* = 0.51). The median household annual income ranged between $50,000 and $74,999. Participants identified themselves mostly as White (72.1%), Hispanic (19.3%), Black (18.3%), and Asian (1.4%).

### Measures and variable selection

#### Independent variables

All independent variables utilized in this study were assessed at baseline and included in the analyses as either continuous or categorical (binary) predictors. They were categorized into three groups: individual characteristics, PTEs and other adverse childhood experiences and characteristics of the social environment.

#### Individual characteristics

In addition to sociodemographic characteristics, the present study included the following variables related to the child: screentime, the *Behavioural Inhibition System and the Behavioural Approach System* (BIS/BAS) [34], prosocial behaviour, and physical activity. Screentime is a composite score that includes watching television, videos (e.g., YouTube), playing video games, texting on a cell phone/tablet/computer, visiting social networking sites, and video chatting. The BIS/BAS questionnaire consists of one BIS scale and three BAS subscales. Participants rated items based on how they typically think or feel. Total values were calculated by adding up all responses for each (sub)scale. Higher BIS and BAS (sub)scale scores indicated greater sensitivity to punishment and reward, respectively. Prosocial behaviour was assessed using a three-item subscale derived from the *Strengths and Difficulties Questionnaire* (SDQ) [35]. The subscale was built using the average item mean scores. Finally, participants were asked how many days they were physically active for a total of at least 60 min per day, and how many days they had done exercises to strengthen or tone their muscles, during the past week [36]. If both questions were answered, the mean value was used. If only one question was answered, that value was used as a measure of physical activity.

#### PTEs and other adverse childhood experiences

PTEs meeting DSM-5 criteria were assessed using the *Kiddie Schedule for Affective Disorders and Schizophrenia Present and Lifetime Version* (K-SADS-PL) [33]. All confirmed PTEs were counted (number of PTEs). Additionally, the PTEs were categorized into poly-victimization, accidents/natural disasters/fire, terrorism/war/community violence, physical abuse, domestic violence, sexual abuse, and traumatic loss of loved ones. The *Parental Monitoring Scale* (PMS) measures caregiver awareness of their child’s whereabouts, both at home and when they are not [37]. Low scores on the PMS may indicate physical neglect [38]. Mean values were computed for each participant and used for analyses. Financial struggles within the immediate family over the past year, including difficulties with food, medical, or other expenses (utilities), or facing eviction due to unpaid rent/mortgage, were also included as binary indicators of physical neglect [38]. The Acceptance subscale from the *Revised Child’s Report of Parental Behaviour Inventory* (CRPBI) was utilized to assess parenting/acceptance by the primary caregiver [39]. This subscale contains items related to comfort, positive emotions, and open communication. Low scores on the CRPBI may suggest emotional neglect [38]. A mean value was computed for analysis purposes. Bullying was evaluated using the K-SADS-PL and categorized as a binary variable [33].

#### Characteristics of the social environment

The t-scores from the *Adult Self-Report* (ASR), which are available in the ABCD dataset, are included as measures of parental externalizing and internalizing problems [40]. These t-scores are standardized scores, obtained by converting raw scores from the ASR questionnaire using a formula that compares them to a large, age- and gender-matched normative sample. When completing the ASR, participants report their behaviour, thoughts, and feelings over the previous six months by rating how applicable the items are. Information about parental alcohol and drug problems was gathered using a modified version of the *Family History Assessment* from the *National Consortium on Alcohol and Neurodevelopment in Adolescence* [41]. If at least one parent had a problem with alcohol or drugs at the time of study admission, the item was coded as one. The Family Conflict subscale from the *Family Environment Scale* (FES) addresses topics such as fighting, anger, criticism, yelling, and loss of temper within the family [42]. Participants were required to indicate whether each item was applicable or not applicable. Scales measuring School Environment, School Involvement and School Disengagement are derived from the *School Risk and Protective Factors* (SRPF) survey [43]. The scores for each item were added up to build scale scores. Additionally, we included the item “My neighbourhood is safe from crime”, coded on a Likert scale ranging from 1 to 5 [43].

#### Dependent variables

The Internalizing and Externalizing Problem scores obtained from the *Brief Problem Monitor youth* form (BPM-Y) served as dependent variables [44]. Participants rated their behaviour, thoughts, and feelings over the previous six months by indicating the applicability of the items. The BPM-Y was completed six months after the baseline assessment for the first time in the ABCD study. All subsequent follow-up assessments up to a 3-year follow-up (six measurement time points in total) were included. A detailed description of the measures is provided in Table 1.

**Table 1:** Measures of predictors and dependent variables in the total sample

Construct	Informant	Measure	Number of items	Min–Max in the current sample/Answer options	M (SD)/n (%)	α	Number of missing data
Individual characteristics							
Child´s age	Caregiver	Demographics	1	8–11 years	9.48 (0.51)		0
Child´s gender (listed on the original birth certificate)	Caregiver	Demographics	1	Male (1) female (0)	2123 (51.3) 2018 (48.7)		0
Child´s race	Caregiver	Demographics	1	White/Non-White	2985 (72.1) 1150 (27.8)		6
Child´s ethnicity	Caregiver	Demographics	1	Hispanic/Non-Hispanic	799 (19.3) 3302 (79.7)		40
Caregiver´s education	Caregiver	Demographics	1	3 (the third grade completed at school)−21 (Doctoral degree)	16.40 (2.56)		1
Caregiver´s employment	Caregiver	Demographics	1	Employed/non-employed	2835 (68.5) 1300 (31.4)		6
Family income (past year)	Caregiver	Demographics	1	1 (< $5,000)−10 (≥ $200,000)	6.80 (2.48)		150
Average screentime (workday)	Child	Youth Screen Time	6	0–24 h	3.85 (3.30)		8
BIS (anticipation of punishment)	Child	BIS/BAS	7	Items: 0–3; Scale: 0–21	9.62 (3.82)	.62	9
BAS: Drive (intensity of goal directed behaviour)	Child	BIS/BAS	4	0–3; 0–12	4.26 (3.11)	.77	9
BAS: Fun Seeking (willingness to approach a potentially rewarding event)	Child	BIS/BAS	4	0–3; 0–12	5.86 (2.72)	.66	9
BAS: Reward Responsiveness (anticipation of reward)	Child	BIS/BAS	5	0–3; 0–15	11.10 (2.94)	.73	9
Prosocial behaviour	Child	SDQ	3	0–2; The subscale was built using the average item mean scores	1.69 (0.37)	.58	19
Physical activity	Child	Risk Behaviour	2	0–7 days	2.69 (1.76)		11
PTEs and other adverse childhood experiences							
PTEs (number)	Caregiver	K-SADS-PL	17	1–17	1.42 (1.06)		0
Poly-victimization (being exposed to at least two different PTEs)	Caregiver	K-SADS-PL	17	Yes/No	1147 (27.7) 2994 (72.3)		0
Accidents/natural disasters/fire	Caregiver	K-SADS-PL	4	Yes/No	1260 (30.4) 2881 (69.6)		0
Terrorism/war/community violence	Caregiver	K-SADS-PL	3	Yes/No	141 (3.4) 4000 (96.6)		0
Physical abuse	Caregiver	K-SADS-PL	3	Yes/No	109 (2.6) 4032 (97.4)		0
Domestic violence	Caregiver	K-SADS-PL	1	Yes/No	929 (22.4) 3212 (77.6)		0
Sexual abuse	Caregiver	K-SADS-PL	3	Yes/No	222 (5.4) 3919 (94.6)		0
Traumatic loss	Caregiver	K-SADS-PL	1	Yes/No	2778 (67.1) 1363 (32.9)		0
Parental monitoring vs. physical neglect	Child	PMS	5	1–5; The subscale was built using the average item mean scores: 1.80–5	4.39 (0.52)	.45	9
Struggling expenses: food	Caregiver	Demographics	1	Yes/No	518 (12.5) 3590 (86.7)		33
Evicted from home	Caregiver	Demographics	1	Yes/No	81 (2.0) 4047 (97.7)		13
Struggling expenses: medical	Caregiver	Demographics	2	Yes/No	673 (16.3) 3451 (83.3)		17
Struggling expenses: other	Caregiver	Demographics	3	Yes/No	881 (21.3) 3231 (78.0)		29
Parenting/acceptance vs. emotional neglect	Child	CRPBI	5	1–3; The subscale was built using the average item mean scores	2.78 (0.29)	.68	13
Bullying	Caregiver	K-SADS-PL	1	Yes/No	871 (21.0) 3267 (78.9)		3
Characteristics of the social environment							
Caregiver: internalizing problems	Caregiver	ASR	39	0–2; t-scores: 30–95	51.01 (10.98)	.92	36
Caregiver: externalizing problems	Caregiver	ASR	35	0–2; t-scores: 30–90	48.20 (9.96)	.87	36
Parents: alcohol/drug problems	Caregiver	Family History	4	Yes/No	1156 (27.9) 2797 (67.5)		188
Family conflicts	Child	FES	9	0–9	2.17 (2.01)	.68	11
School: environment	Child	SRPF	6	1–4; 6–24	19.89 (2.91)	.61	10
School: involvement	Child	SRPF	4	1–4; 4–16	13.03 (2.42)	.65	10
School: disengagement	Child	SRPF	2	1–4; 2–8	3.80 (1.50)		10
Neighbourhood	Child	Toolkit	1	1–5	3.93 (1.15)		11
Dependent variables							
Internalizing problems	Child	BPM-Y	6	0–2; 0–12	1.98 (2.11)	.70	
Externalizing problems	Child	BPM-Y	7	0–2; 0–14	2.13 (2.07)	.68	

*N* = 4,141; Cronbach’s alpha (α ≥.9: excellent,.8 ≤ α <.9: good,.7 ≤ α <.8: acceptable,.6 ≤ α <.7: questionable,.5 ≤ α <.6: poor, α <.5: unacceptable), a measure of internal consistency or reliability for a test or scale was calculated for all scales containing at least three items at baseline for all measures, except for BPM-Y. All data reported for BPM-Y are from the 6-month follow-up assessment; T-scores are standardized scores that compare an individual’s performance to a representative sample of their age and gender group. They have a standard mean of 50 and a standard deviation of 10, allowing for consistent interpretation across various scales. A t-score of 50 is considered average, while scores above 65 are often deemed clinically significant; *BIS/BAS* the Behavioural Inhibition System and the Behavioural Approach System; *SDQ* the Strengths and Difficulties Questionnaire; *PTEs* Potentially Traumatic Events; *K-SADS-PL* the Kiddie Schedule for Affective Disorders and Schizophrenia Present and Lifetime Version; *PMS* The Parental Monitoring Scale; *CRPBI* the Revised Child’s Report of Parental Behaviour Inventory; *ASR* the Adult Self Report; *FES* the Family Environment Scale; *SRPF* the School Risk and Protective Factors; *BPM-Y* the Brief Problem Monitor youth form

### Statistical analysis

Statistical analyses were conducted using IBM SPSS Statistics 29, R Version 4.4.0, and Python 3.10.11, utilizing the *lcmm* [45] and *Scikit-learn* [46] packages. The significance levels for all statistical tests were set to *p* < 0.05 (two-tailed).

Data on the two dependent variables, assessed at six time points using the BPM-Y, were missing completely at random (MCAR; Little’s MCAR test: *ꭓ*^*2*^(35) = 0.10, *p* = 1.000; 9.0% missing data). Degrees of freedom for this test were determined using the IBM SPSS Statistics (Version 29; IBM Corp., Armonk, NY, USA) software package. This allowed us to impute the data using expectation–maximization [47] and include all 4,141 participants in *Latent Growth Mixture Modelling* (LGMM).

To ensure a clear differentiation between symptom domains, two independent LGMMs were estimated: the first model focused exclusively on trajectories of internalizing problems, while the second, separate model focused on externalizing problems across all measurement time points. Intercepts and slopes were examined as random effects using the class-specific variance–covariance matrix of the random effects. Quadratic parameters were included in both the fixed effects and latent class-specific mixture effects to capture non-linear growth trajectories. These terms significantly improved the model fit. To ensure model convergence and avoid local maxima, a grid search procedure with 100 random starts was employed. Model solutions ranging from one to five classes were compared using model fit indices: the Akaike Information Criterion (AIC), Bayesian Information Criterion (BIC), Sample-Size Adjusted BIC (SSABIC), entropy, Lo-Mendell-Rubin Likelihood Ratio Test (LMR-LRT), and the Bootstrap Likelihood Ratio Test (BLRT with 500 resamples). To ensure reproducibility and transparency, the analytical code has been made available as Supplementary Material 6. This includes the R syntax for the LGMMs, covering model estimation via grid search, fit index calculation, and the BLRT for internalizing trajectories; the same analytical procedure was applied to the externalizing problems models. The determination of the number of trajectory classes in LGMM has been a topic of debate among researchers, with the compromise between substantive justification, interpretability, theoretical robustness and model fit being crucial. While fit indices are relative, entropy values provide an absolute measure of classification certainty. Therefore, when fit statistics are similar, models with higher entropy should be preferred [48, 49]. Many theorists in this field agree that both model construction and final model selections should be based on theoretical justification and interpretability of the results [50–53]. The most informative models are those that align with theory or prior research [54]. Therefore, our final decision was not solely based on statistical parameters, but also on alignment with prior research. Once the optimal number of trajectory classes was determined, participants were assigned to classes based on posterior class probabilities to ensure that each participant was placed in the most probable latent class identified by the LGMM.

ML predictive modeling was used to identify predictors of membership in the chronic problems vs. resilient trajectory groups. In accordance with previous research, both trajectories were expected to be derived from LGMM [6]. Only participants assigned to either the chronic problems or resilient trajectory group were included in ML analyses, due to high clinical relevance of these two trajectories.

In the first step, a Pearson correlation matrix was inspected for the 37 candidate predictors listed in Table 1. None of the absolute pairwise correlations exceeded .65. Only two pairs exceeded .60 and three others exceeded .50, indicating that strong multicollinearity (≈.80) is absent. To further guard against redundant variables Sequential Forward Feature Selection (SFS) within the cross-validation workflow was incorporated. SFS is now used to select the optimal subset of features during each hyper‑parameter optimization trial, and the final model is trained on this globally‑selected set. Because the correlation analysis showed only modest inter‑feature dependencies, no additional decorrelation technique (e.g., Variance Inflation Factor filtering) was required.

Random Forest and Logistic Regression emerged as the best-performing models, with eXtreme Gradient Boosting (XGBoost) also performing competitively for both targets. All 37 predictors shown in Table 1 were considered. Separate models were developed for internalizing and externalizing problems, each treated as a binary classification task. All preprocessing, feature selection, and model evaluation were performed independently for each target to avoid information leakage. A detailed description of the ML pipeline can be found in the Supplementary Material (see Supplementary Text 1). Additionally, the complete analytical code—including data preparation, exploratory model cross-validation, nested cross-validation, feature selection, SHAP values, confusion matrices, and supplemental statistical tests—has been made available as Supplementary Material 7.

The *SHapley Additive exPlanations* (SHAP) bee-swarm plots summarize the influence of each feature on trajectory prediction for both internalizing and externalizing problems. SHAP values capture feature contributions across all predictions, including non-linear and interaction effects. Rows represent predictors ordered by mean absolute SHAP value; higher values indicate greater importance. SHAP values may be positive or negative, showing the direction and distribution of each predictor’s influence. Each point represents a SHAP value, with density indicated by stacking and color denoting predictor magnitude (deep red = high, light blue = low). Points with similar SHAP values may be jittered along the y-axis to reveal distribution density [55].

Mann–Whitney U tests, Common Language Effect Size (CLES), and Rank-Biserial Correlation (RBC) were incorporated as complementary validation measures for the ML results [56]. Together, these statistical measures validated the SFS and SHAP results, to ensure that the most influential predictors identified by the ML pipeline also exhibit strong and consistent statistical differences between the trajectory groups. Specifically:

1. Mann–Whitney U tests were used to compare the distributions of each selected feature between the resilient and chronic problems groups. This nonparametric test assesses whether the feature values differ systematically between the two trajectory groups, providing a robust validation of the ML-based feature selection.
2. CLES quantifies the probability that a randomly selected observation from the chronic-problems group will have a higher feature value than a randomly selected observation from the resilient group. This measure complements the U test by providing an interpretable effect size for the observed differences.
3. RBC further characterizes the strength and direction of the association between each feature and group membership, offering a standardized effect size that aligns with the rank-based nature of the Mann–Whitney U test.

## Results

### Identification of internalizing and externalizing problem trajectories

**Fig. 1:**
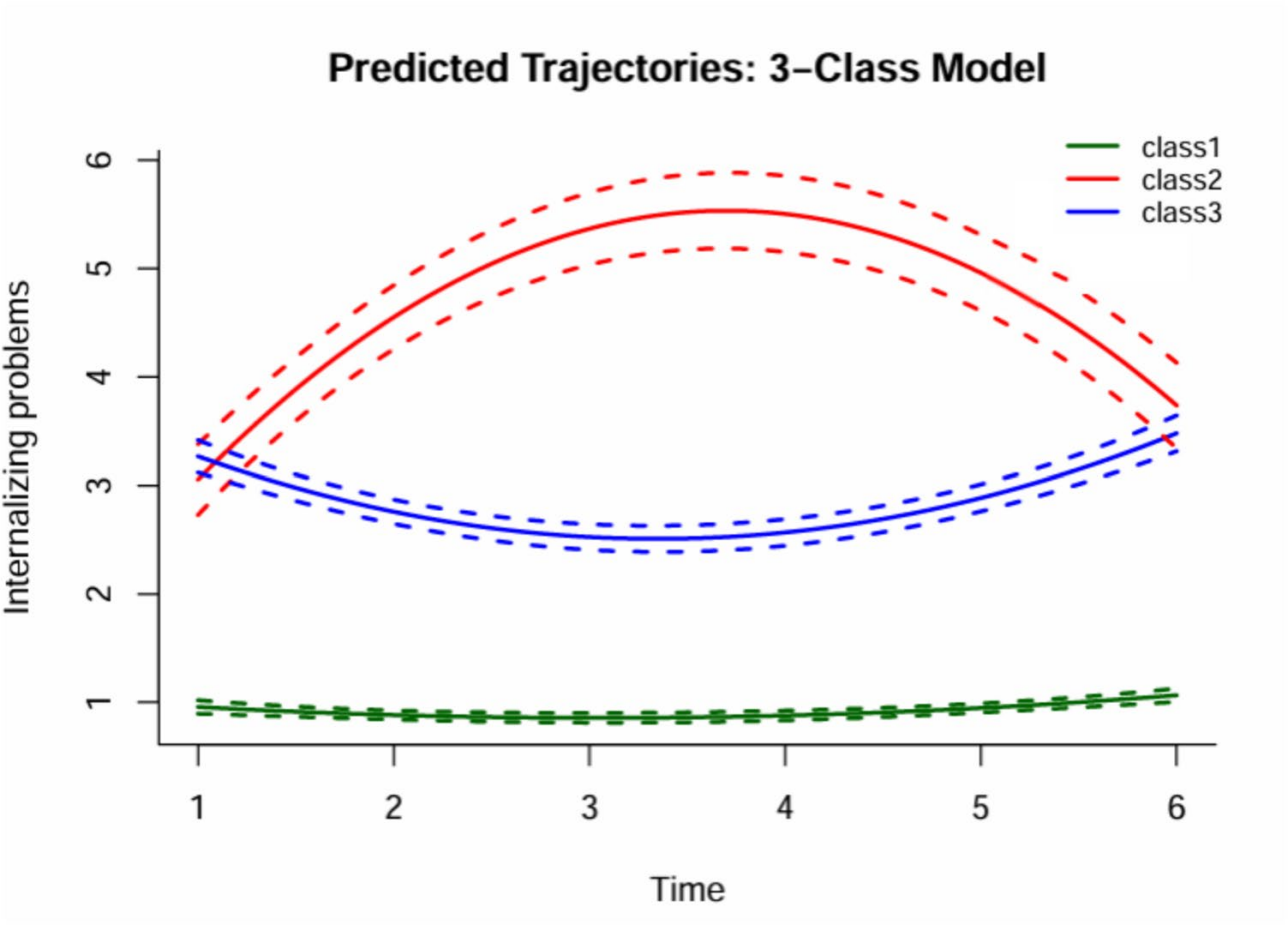
Internalizing problems: The observed means and 95% confidence intervals of the three trajectories across the six assessments

The fit indices for various class solutions are provided in Supplementary Table 2. For internalizing problems, a 3-class model was chosen. Although AIC, BIC, SABIC and LMR-LRT indicated a better fit for the 4-class model, entropy indicated that the 3-class model provided the best overall fit to the data. The non-convergence of the BLRT when comparing the three- and four-class solutions (indicated by a NaN result) serves as a diagnostic indicator of local maxima and convergence instability [57]. This failure suggests that the 4-class model is empirically under-identified and that the additional complexity is not supported by the data. Consistent with this finding, the 4-class model indicated moderate, mild, and two very similar trajectories characterized by low levels of problems. Since those two trajectories seemed redundant and were not of any particular theoretical interest, the 3-class model was retained instead. The three identified trajectories were labelled as follows: 1. “Resilient” (*n* = 2,427, 58.6% of the sample), characterized by low internalizing problems (*b* = 1.08, *SE* = 0.06, *p* < 0.001) that slightly changed over time but still remained minimal. The “Resilient” trajectory exhibited a significant negative linear slope (*b* = −0.15, *SE* = 0.04, *p* < 0.001) and a significant positive quadratic term (*b* = 0.02, *SE* = 0.01, *p* < 0.001); 2. The “Mild” trajectory (*n* = 1,373, 33.2%) was characterized by initially mild problems (*b* = 4.05, *SE* = 0.13, *p* < 0.001). This trajectory exhibited a significant negative linear slope (*b* = −0.92, *SE* = 0.08, *p* < 0.001) and a significant positive quadratic term (*b* = 0.14, *SE* = 0.01, *p* < 0.001), indicating a decelerating decline over the observed time points.; and 3. The “Moderate chronic” trajectory (*n* = 341, 8.2%) was characterized by relatively low problems at baseline (*b* = 0.88, *SE* = 0.27, *p* = 0.001) that significantly worsened over time, as indicated by a significant positive linear slope (*b* = 2.52, *SE* = 0.17, *p* < 0.001) and a significant negative quadratic term (*b* = −0.34, *SE* = 0.02, *p* < 0.001), indicating a decelerating increase over the observed time points. The mean posterior probabilities were adequate for the “Resilient” (.89), “Mild” (.85), and “Moderate chronic” (.82) trajectories. The observed means and 95% CIs of the three trajectories across the six assessments are presented in Fig. 1.

**Fig. 2:**
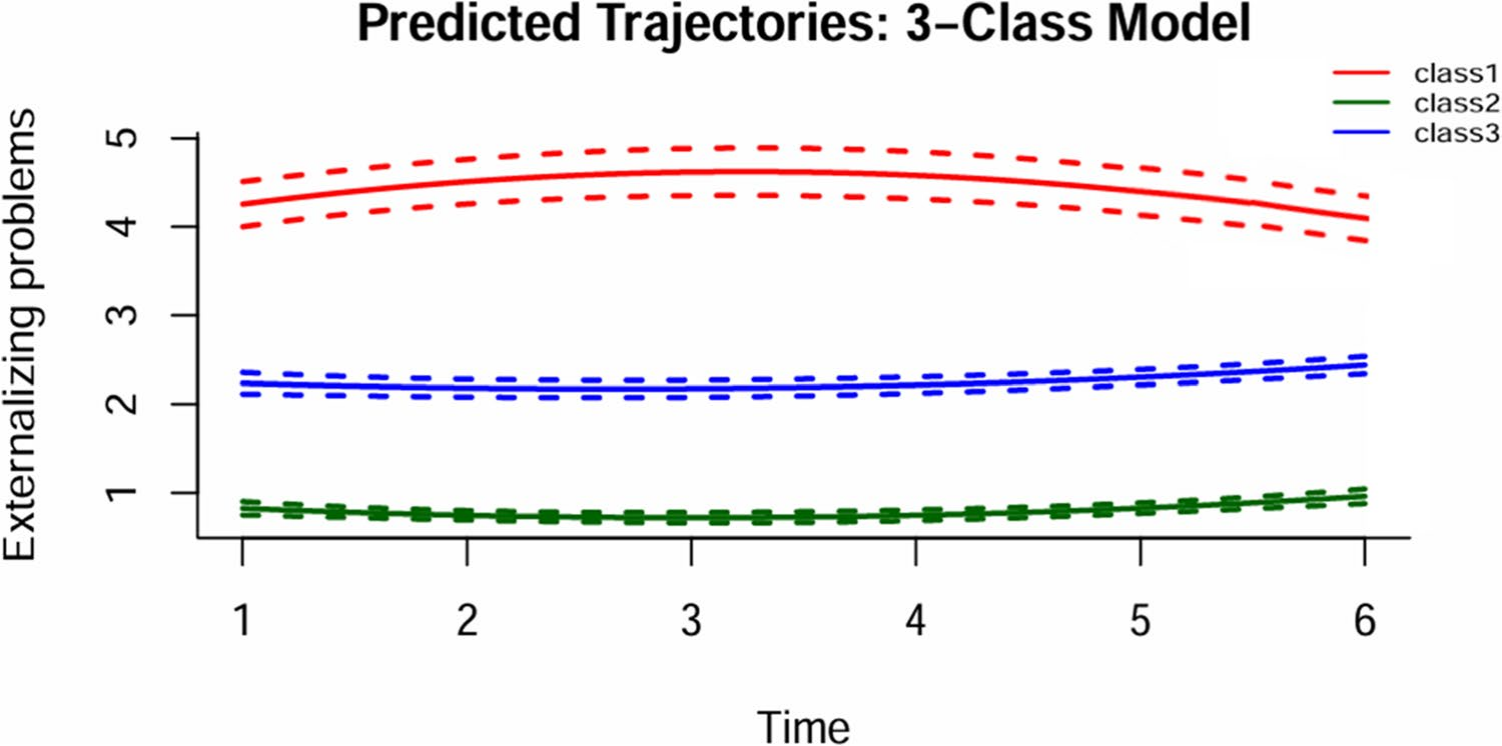
Externalizing problems: The observed means and 95% confidence intervals of the three trajectories across the six assessments

**Fig. 3:**
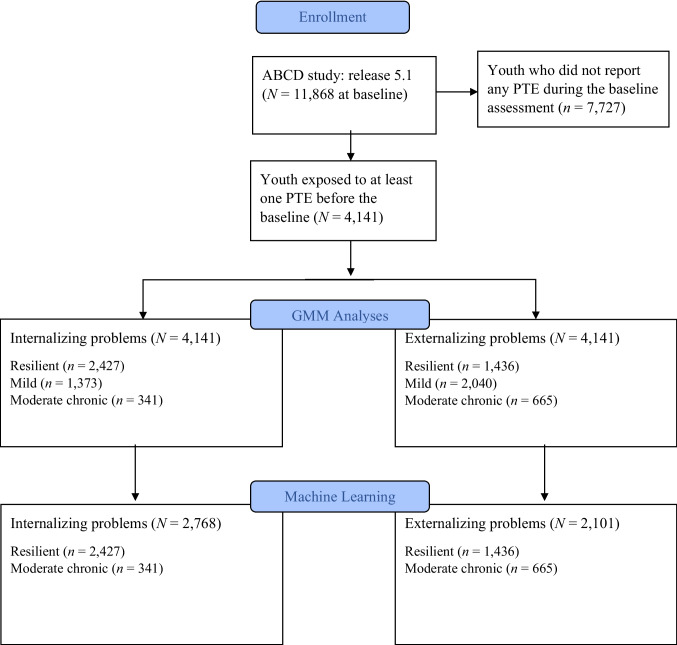
CONSORT flowchart of study participants. *Note*. PTEs = Potentially Traumatic Events; LGMM = Latent Growth Mixture Modelling

For externalizing problems, most model fit indices pointed towards choosing between the 4-class model and the 5-class model. LMR-LRT did not show a preference for any of the solutions. However, the 4-class model indicated resilient, moderate, and two very similar trajectories characterized by mild problem levels. The BLRT was conducted to compare the 3- and 4-class solutions; however, the 4-class model failed to converge during the bootstrapping procedure. This lack of convergence suggests that the 4-class model is empirically under-identified. The derived 3-class model is easily interpretable and was ultimately retained. The trajectories were labelled as follows: 1. “Resilient” (*n* = 1,436, 34.7%) was characterized by initially low externalizing problems (*b* = 0.96, *SE* = 0.07, *p* < 0.001) that significantly changed over time but remained minimal. The “Resilient” trajectory exhibited a significant negative linear slope (*b* = −0.16, *SE* = 0.05, *p* < 0.001) and a significant positive quadratic term (*b* = 0.03, *SE* = 0.01, *p* < 0.001), representing a decelerating decrease in a U-shaped pattern; 2. The “Mild” trajectory (*n* = 2,040, 49.3%) was characterized by mild problems at baseline (*b* = 2.34, *SE* = 0.09, *p* < 0.001). Similar to the “Resilient” group, this trajectory followed a decelerating decrease in a U-shaped pattern, with a significant negative linear slope (*b* = −0.13, *SE* = 0.05, *p* = 0.006) and a significant positive quadratic term (*b* = 0.02, *SE* = 0.01, *p* < 0.001); 3. The “Moderate chronic” trajectory (*n* = 665, 16.1%) was characterized by mild problems at baseline (*b* = 3.85, *SE* = 0.17, *p* < 0.001). This group followed an inverted U-shaped pattern, with a significant positive linear slope (*b* = 0.48, *SE* = 0.10, *p* < 0.001) and a significant negative quadratic term (*b* = −0.07, *SE* = 0.01, *p* < 0.001). The mean posterior probabilities were adequate for “Resilient” (.83), “Mild” (.81), and “Moderate chronic” (.88) trajectories. The observed means of the three trajectories of externalizing problems across time are presented in Fig. 2. Figure 3 illustrates the flow of participants in the study.

### Predictors of internalizing and externalizing problem trajectories

**Fig. 4:**
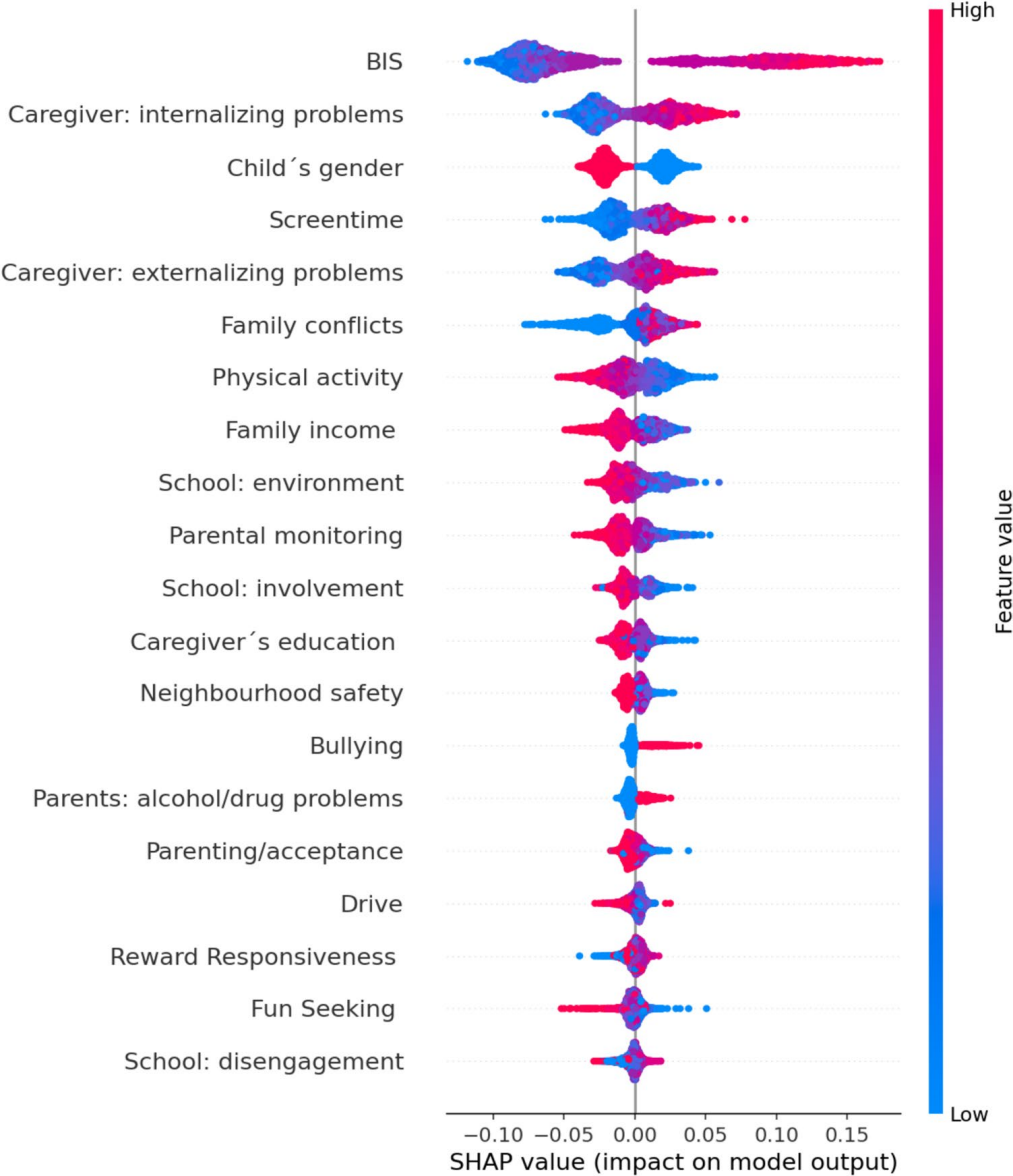
Internalizing problems: SHAP Summary Bee Swarm plot. *Note*. SHAP values for predictors in the machine learning model used to classify between the “Moderate chronic” and the “Resilient” trajectories of internalizing problems. Positive values of the red bars indicate predictors contributing to the “Moderate chronic” trajectory, while negative values of the red bars indicate predictors contributing to the “Resilient” trajectory. SHAP = SHapley Additive exPlanations; BIS = the Behavioural Inhibition System

The Random Forest classifier achieved the highest mean discriminative performance for the internalizing problems trajectory (F1_macro = .622 ± .024; Matthew’s correlation coefficient (MCC) = .250 ± .024; Area Under the Curve (AUC) = .715 ± .030). XGBoost produced a numerically lower result (F1_macro = .608 ± .024, MCC = .219 ± .024, and AUC = .674 ± .032), yielding an absolute macro-F1 difference of 0.014 in favor of Random Forest. SFS and SHAP analysis of the final model indicated that BIS, caregiver´s mental health problems, gender, screen time, family conflicts, physical activity, family income, perceiving school as a resource, and parental monitoring were the most influential predictors (listed in descending order of mean |SHAP|). The “Moderate chronic” trajectory was characterized by a distinct feature set, where pronounced BIS, caregiver mental health problems, and female gender served as primary indicators. Additional predictive weight was attributed to a constellation of behavioral and environmental variables, specifically higher screentime, family conflicts, and lower physical activity, alongside lower family income, negative school attitudes, and reduced parental monitoring (indicative of physical neglect). On the contrary, lower endorsement of BIS, having parents with fewer mental health problems, being male, reporting lower usage of screen media, experiencing fewer family conflicts, engaging in regular physical activity, having a higher family income, positive attitudes toward school, and receiving more parental monitoring all contributed to resilience. SHAP values for the top 20 predictors are presented in Fig. 4. The SFS features for a model that involves internalizing problems are provided in Supplementary Table 3. Statistical analyses (Mann–Whitney U, CLES, RBC) confirmed the significance of these predictors (see Table 2).

**Table 2:** Sample description including comparison between resilient and moderate chronic groups regarding all predictors included in the machine learning models for internalizing and externalizing problems

	Internalizing problems			Externalizing problems		
Construct	Resilient (*n* = 2427): M (SD)/n (%)	Moderate chronic (*n* = 341): M (SD)/n (%)	Group Comparison: *U*, *p*, CLES, RBC	Resilient (*n* = 1436): M (SD)/n (%)	Moderate chronic (*n* = 665): M (SD)/n (%)	Group Comparison: *U*, *p*, CLES, RBC
Child´s age	9.49 (0.51)	9.47 (0.50)	*U* = 404435.50, *p* =.435,.51,.02	9.49 (0.51)	9.48 (0.51)	*U* = 475935.50, *p* =.891,.51,.00
Child´s gender: female, male	1067 (44.0), 1360 (56.0)	204 (59.8), 137 (40.2)	*U* = 348173.00, ***p* <.001**,.59,.16	738 (51.4), 698 (48.6)	290 (43.6), 375 (56.4)	*U* = 440305.00, ***p* <.001**,.54, -.08
Child´s race: white, non-white	1771 (73.0), 652 (26.9)	245 (71.8), 95 (27.9)	*U* = 407657.50, *p* =.688,.51,.01	1080 (75.2), 353 (24.6)	460 (69.2), 205 (30.8)	*U* = 446962.50, ***p* =.003**,.54,.06
Child´s ethnicity: Hispanic, non-Hispanic	432 (17.8), 1969 (81.1)	79 (23.2), 257 (75.4)	*U* = 381104.50, ***p* =.015**,.54, -.06	264 (18.4), 1156 (80.5)	141 (21.2), 519 (78.0)	*U* = 455610.00, *p* =.137,.51, -.03
Caregiver´s education level	16.62 (2.50)	15.97 (2.77)	*U* = 357668.00, ***p* <.001**,.57,.14	16.96 (2.40)	15.84 (2.59)	*U* = 342572.00, ***p* <.001**,.63,.28
Caregiver´s employment: working, non-working	1682 (69.3), 741 (30.5)	221 (64.8), 119 (34.9)	*U* = 393711.50, *p* =.099,.52,.04	1031 (71.8). 404 (28.1)	429 (64.5), 235 (35.3)	*U* = 441935.50, ***p* <.001**,.54,.07
Family income for the past 12 months	6.99 (2.44)	6.31 (2.54)	*U* = 321688.00, ***p* <.001**,.58,.17	7.40 (2.22)	6.10 (2.55)	*U* = 304459.00, ***p* <.001**,.65,.31
Average screentime on a workday	3.50 (3.07)	4.51 (3.66)	*U* = 339046.50, ***p* <.001**,.59, -.18	2.98 (2.56)	5.10 (4.03)	*U* = 316518.50, ***p* <.001**,.69, -.34
BIS	8.80 (3.60)	11.44 (4.05)	*U* = 256402.50, ***p* <.001**,.70, -.38	9.01 (3.72)	10.48 (4.14)	*U* = 377849.00, ***p* <.001**,.61, -.21
BAS: Drive	4.16 (3.07)	4.37 (3.20)	*U* = 399477.00, *p* =.326,.52, -.03	3.74 (2.91)	5.18 (3.32)	*U* = 353845.00, ***p* <.001**,.63, -.26
BAS: Fun Seeking	5.72 (2.71)	5.82 (2.82)	*U* = 401439.50, *p* =.401,.51, -.03	5.34 (2.56)	6.54 (2.94)	*U* = 362139.00, ***p* <.001**,.62, -.24
BAS: Reward Responsiveness	10.97 (2.97)	11.29 (2.90)	*U* = 386668.00, *p* =.055,.53, -.06	10.76 (2.94)	11.66 (2.95)	*U* = 887447.50, ***p* <.001**,.59, -.19
Prosocial behaviour	1.69 (0.36)	1.69 (0.37)	*U* = 407197.50, *p* =.713,.51,.01	1.75 (0.32)	1.57 (0.42)	*U* = 358585.50, ***p* <.001**,.64,.24
Physical activity	2.77 (1.75)	2.34 (1.68)	*U* = 352422.50, ***p* <.001**,.57,.15	2.82 (1.73)	2.58 (1.81)	*U* = 432257.50, ***p* <.001**,.54,.09
Number of PTEs	1.40 (1.14)	1.55 (1.53)	*U* = 385611.50, ***p* =.008**,.54, -.07	1.31 (0.99)	1.61 (1.22)	*U* = 392331.50, ***p* <.001**,.58, -.18
Poly-victimization: yes, no	614 (25.3), 1813 (74.7)	110 (32.3), 231 (67.7)	*U* = 385005.50, ***p* =.006**,.55, -.07	302 (21.0), 1134 (79.0)	252 (37.9), 413 (62.1)	*U* = 396949.00, ***p* <.001**,.61, -.17
Accidents/natural disasters/fire: yes, no	732 (30.2), 1695 (69.8)	94 (27.6), 247 (72.4)	*U* = 403066.50, *p* =.327,.51,.03	424 (29.5), 1012 (70.5)	208 (31.3), 457 (68.7)	*U* = 469106.00, *p* =.416,.51, -.02
Terrorism/war/community violence: yes, no	92 (3.8), 2335 (96.2)	14 (4.1), 327 (95.9)	*U* = 412500.50, *p* =.777,.50,.00	40 (2.8), 1396 (97.2)	29 (4.4). 636 (95.6)	*U* = 469948.00, *p* =.060,.52, -.02
Physical abuse: yes, no	56 (2.3), 2371 (97.7)	13 (3.8), 328 (96.2)	*U* = 407576.00, *p* =.095,.54, -.02	21 (1.5), 1415 (98.5)	32 (4.8), 633 (95.2)	*U* = 461476.50, ***p* <.001**,.57, -.03
Domestic violence: yes, no	472 (19.4), 1955 (80.6)	99 (29.0), 242 (71.0)	*U* = 374143.00, ***p* <.001**,.57, -.10	225 (15.7), 1211 (84.3)	217 (32.6), 448 (67.4)	*U* = 396476.50, ***p* <.001**,.62, -.17
Sexual abuse: yes, no	120 (4.9), 2307 (95.1)	27 (7.9), 314 (92.1)	*U* = 401499.00, ***p* =.022**,.54, -.03	65 (4.5), 1371 (95.5)	54 (8.1), 611 (91.9)	*U* = 460310.50, ***p* <.001**,.54, -.04
Traumatic loss: yes, no	1637 (67.4), 790 (32.6)	224 (65.7), 117 (34.3)	*U* = 406519.00, *p* =.517,.51, -.02	978 (68.1), 458 (31.9)	435 (65.4), 230 (34.6)	*U* = 464615.00, *p* =.221,.52,.03
Parental monitoring	4.44 (0.48)	4.29 (0.56)	*U* = 344632.00, ***p* <.001**,.59,.17	4.49 (0.45)	4.22 (0.57)	*U* = 334454.00, ***p* <.001**,.65,.30
Struggling expenses: food: yes, no	248 (10.2), 2158 (88.9)	56 (16.4), 282 (82.7)	*U* = 381158.00, ***p* <.001**,.56, -.06	106 (7.4), 1319 (91.9)	134 (20.2), 528 (79.4)	*U* = 411286.00, ***p* <.001**,.62, -.13
Evicted from home: yes, no	39 (1.6), 2381 (98.1)	12 (3.5), 326 (95.6)	*U* = 401051.00, ***p* =.013,**.54, -.02	11 (0.8), 1423 (99.1)	18 (2.7), 645 (97.0)	*U* = 466111.50, ***p* <.001**,.55, -.02
Struggling expenses: medical: yes, no	333 (13.7), 2085 (85.9)	88 (25.8), 252 (73.9)	*U* = 361278.00, ***p* <.001**,.59, -.12	151 (10.5), 1280 (89.1)	159 (23.9), 504 (75.8)	*U* = 410668.50, ***p* <.001**,.60, -.13
Struggling expenses: other: yes, no	430 (17.7), 1984 (81.7)	90 (26.4), 247 (72.4)	*U* = 370584.00, ***p* <.001**,.57, -.09	184 (12.8), 1244 (86.6)	205 (30.8), 456 (68.6)	*U* = 386396.00, ***p* <.001**,.63, -.16
Parenting/acceptance	2.80 (0.28)	2.74 (0.33)	*U* = 367287.00, ***p* <.001**,.56,.11	2.84 (0.26)	2.71 (0.35)	*U* = 361976.00, ***p* <.001**,.63,.24
Bullying: yes, no	409 (16.9), 2016 (83.1)	104 (30.5), 237 (69.5)	*U* = 357097.00, ***p* <.001**,.59, -.14	202 (14.1), 1232 (85.8)	222 (33.4), 443 (66.6)	*U* = 384796.00, ***p* <.001**,.63, -.19
Caregiver: internalizing problems	49.78 (10.67)	53.82 (11.04)	*U* = 317921.00, ***p* <.001**,.61, -.22	48.55 (10.22)	54.00 (11.23)	*U* = 335491.50, ***p* <.001**,.64, -.29
Caregiver: externalizing problems	47.24 (9.64)	50.10 (9.98)	*U* = 334990.50, ***p* <.001**,.58, -.17	45.88 (9.48)	51.21 (10.00)	*U* = 324070.50, ***p* <.001**,.65, -.31
Parents: alcohol/drug problems: yes, no	611 (25.2), 1709 (70.4)	123 (36.1), 206 (60.4)	*U* = 339469.50, ***p* <.001**,.57, −11	318 (22.1), 1054 (73.4)	227 (34.1), 410 (61.7)	*U* = 382543.00, ***p* <.001**,.58, -.12
Family conflicts	1.89 (1.92)	2.57 (2.09)	*U* = 330251.50, ***p* <.001**,.60, -.20	1.54 (1.70)	3.16 (2.24)	*U* = 270189.00, ***p* <.001**,.73, -.43
School: environment	20.08 (2.84)	19.31 (3.13)	*U* = 351341.50, ***p* <.001**,.58,.15	20.39 (2.61)	19.08 (3.33)	*U* = 365969.00, ***p* <.001**,.63,.23
School: involvement	13.21 (2.32)	12.60 (2.68)	*U* = 362270.00, ***p* <.001**,.57,.12	13.47 (2.11)	12.21 (2.78)	*U* = 352063.00, ***p* <.001**,.65,.26
School: disengagement	3.72 (1.45)	3.88 (1.51)	*U* = 388542.00, *p* =.070,.53, -.06	3.52 (1.34)	4.33 (1.65)	*U* = 339934.00, ***p* <.001**,.65, -.29
Neighbourhood: safety from crime	4.05 (1.09)	3.78 (1.20)	*U* = 361359.00, ***p* <.001**,.57,.12	4.13 (1.04)	3.61 (1.30)	*U* = 370354.00, ***p* <.001**,.63,.22

*BIS* the Behavioural Inhibition System; *BAS* the Behavioural Approach System; *PTEs* Potentially Traumatic Events; *U* Mann–Whitney U test; *CLES* the Common language effect size; *RBC* and Rank-biserial correlation; Significant *p*-values are indicated in bold

**Fig. 5:**
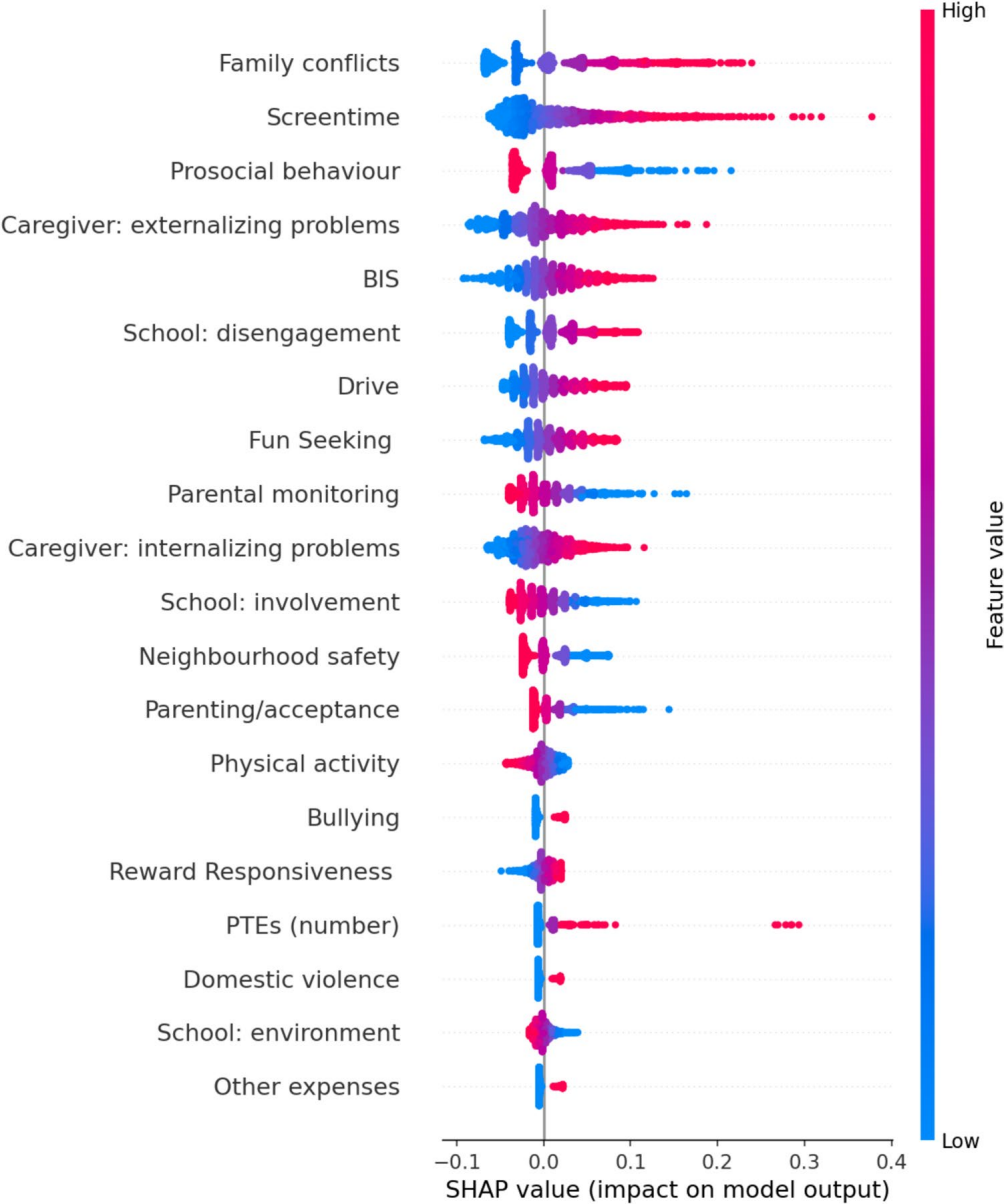
Externalizing problems: SHAP Summary Bee Swarm plot. *Note*. SHAP values for predictors in the machine learning model used to classify between the “Moderate chronic” problems and the “Resilient” trajectories of externalizing problems. Positive values of the red bars indicate predictors contributing to the “Moderate chronic” trajectory, while negative values of the red bars indicate predictors contributing to the “Resilient” trajectory. SHAP = SHapley Additive exPlanations; BIS = the Behavioural Inhibition System; PTEs = Potentially Traumatic Events

The logistic regression classifier attained the highest mean discriminative performance for the externalizing problems trajectory (F1_macro = .739 ± .020; MCC = .486 ± .039; AUC = .828 ± .019). Support Vector Machine (SVM) performed similarly (F1_macro = .736 ± .020; MCC = .480 ± .039; AUC = .826 ± .019), with an absolute F1 difference of .003 in favor of logistic regression. SFS and SHAP analysis identified family conflicts, screentime, prosocial behaviour, caregiver mental health problems, BIS, school disengagement, BAS (especially Drive and Fun Seeking), and parental monitoring as the most influential predictors (in descending order of mean |SHAP|). Exposure to family conflicts, increased screen media usage, decreased levels of prosocial behaviour, mental health problems in caregivers, high levels of BIS and BAS endorsement, school disengagement, and inadequate parental monitoring all contributed to the “Moderate chronic” trajectory. On the other hand, a family environment free of conflicts, reduced screen media usage, prosocial behaviour, fewer parental mental health issues, lower levels of BIS and BAS, lower scores on the School Disengagement scale, and parental monitoring were all associated with a higher likelihood of being classified as resilient. SHAP values for the top 20 predictors are presented in Fig. 5. The SFS features for a model that involves externalizing problems are provided in Supplementary Table 4. Statistical testing further supported these findings, as demonstrated in Table 2.

## Discussion

The present study aimed to investigate the courses of adjustment following PTEs and to identify the most important factors contributing to “Resilient” and “Moderate chronic” trajectories, utilizing a representative community sample derived from the ABCD study [30].

### Mental health trajectories

Concerning internalizing problems, most youth followed a “Resilient” trajectory, demonstrating healthy functioning across the six assessments. This aligns with a previous study on posttraumatic stress symptoms in children/adolescents who experienced childhood sexual abuse, as well as a meta-analysis including various childhood PTEs [6, 7]. The percentage of trauma-exposed individuals following a chronic trajectory in the meta-analysis aligns with our results. The increasing trend of the “Moderate chronic” trajectory in our study may be due to age effects. The onset of adolescence, which can bring reduced well-being and increased vulnerability to internalizing mental health problems, may contribute to this rise [58]. This trend could also be a result of ongoing stressors over time, as seen in previous research and exacerbated by the COVID-19 pandemic [6, 30, 32]. In contrast to the commonly identified trajectories of recovery and delayed onset, the third trajectory of internalizing problems in our study was “Mild”, reflecting, on average, subthreshold clinical symptoms. Due to the lack of information regarding the timing of PTEs, and considering that youth exposed to PTEs often show significant natural recovery within the first few months following trauma [59], one possible explanation for this discrepancy could be that the events occurred a considerable time ago. Our study may not have captured the early stages of trauma adjustment, which could involve recovery and delayed symptom onset. For example, Meiser-Stedman et al. [59] studied the trajectories of trauma adjustment following single incident traumas in youth. They found that 7.2% of their sample recovered within 9 weeks post-trauma, while only 2.9% showed delayed onset. Recovery may be confused with resilience or a chronic trajectory, depending on the assessment time [6]. Furthermore, delayed onset was the least frequently observed trajectory in the meta-analysis. When identified, it had low prevalence rates and was associated with cumulative stressors. The delayed-onset trajectory had the lowest prevalence within the children´s sample [6]. Furthermore, the differing findings may be due to the fact that we focused on internalizing problems, which may offer a broader perspective on mental health, rather than solely examining trauma adjustment.

It is noteworthy that all three trajectories of externalizing problems show relatively low average means, which may be the result of a tendency for children/adolescents to underestimate their level of externalizing problems as shown in previous research [60]. Furthermore, internalizing problems, such as depression or anxiety, have been more frequently associated with trauma exposure than externalizing problems [20].

### Predictors of the “resilient” versus “moderate chronic” trajectories

Psychological characteristics, increased screentime, sociodemographic variables, family, social environment, and physical activity were identified as the most influential predictors of trajectories for both internalizing and externalizing problems. These findings align with a meta-analysis that identified psychological, environmental, individual and social characteristics, as the most frequently identified predictors of outcome trajectories [6]. Regarding PTE characteristics, the meta-analysis found mixed results, such as high rates of resilience among samples with high trauma exposure, leading to the conclusion that the severity and types of PTEs are not key contributors to the trajectories [6].

The substantial contribution of BIS is not surprising, given the strong positive associations between BIS sensitivity and various psychological difficulties, including anxiety, depression, stress sensitivity, and emotion dysregulation [61].

However, the identification of screentime as one of the most important features of “Moderate chronic” trajectories is remarkable, considering the inconsistent findings of meta-analyses and reviews on the relationship between screentime and youth mental health [62]. One recent study in U.S. adolescents found an association between elevated smartphone use and escalating symptom trajectories, which aligns with our findings [63]. Furthermore, Nagata and colleagues found an association between spending more time on social media and experiencing symptoms of depression later on [64]. Exposure to PTEs, especially cumulative stressors, can facilitate problematic social media use as a coping mechanism and, in turn, foster mental health problems [65]. Research on adolescents exposed to PTEs has shown that they are more likely to engage in high-risk internet and social media activities, e.g., heightened usage, encountering sexual content and being victims of cyberbullying, which can significantly impact their well-being [7, 19]. Further clarification is needed on the intricate relationship between PTEs and screentime, and how they affect youth´s mental health. It is crucial to take into account factors such as excessive and problematic screen media usage, use of various types of media, specific online behaviours, motivations for use, household rules regarding screen time, the screen habits of other family members, and experiences of online victimization.

The predominance of girls in the “Moderate chronic” internalizing problems trajectory is consistent with previous research that has found females, particularly during adolescence, to be more vulnerable to developing trauma-related symptoms and internalizing problems [2, 4, 8–11, 58].

Consistent with existing literature, various family factors such as family conflicts, parental mental health, acceptance, and monitoring appear to play a significant role in trauma adjustment. A child´s exposure to PTEs may trigger trauma-related symptoms in their caregivers, impairing their ability to effectively address the child´s needs [16]. These symptoms can negatively impact parenting strategies (e.g., becoming overprotective), contribute to the development of unhelpful trauma-related beliefs (e.g., blaming the child for the PTE), and result in maladaptive coping mechanisms (e.g., avoidance) [17]. Research on parent–child relationships in the face of adversity has shown that children’s symptoms can influence parental symptoms and vice versa. The bidirectional influences, shared genetic and environmental factors, parental role as a caregiver and role model, as well as parental history of PTEs and their involvement in the current PTE need to be considered when addressing childhood trauma [16, 17].

PTEs and other adversities, although playing a smaller role compared to individual and environmental factors in differentiating between the two trajectories, increased the likelihood of following the “Moderate chronic” trajectory, aligning with expectations based on the current literature [2, 4, 6, 8–10].

In summary, the results indicated that features representing more severe PTEs and heightened psychosocial problems increase the likelihood of classification into “Moderate chronic” trajectories.

## Limitations

Information on PTEs was collected through caregiver reports, which raises the possibility that caregivers may not have disclosed all PTEs the child experienced or that their own history of PTEs may have influenced their reporting. The history of PTEs was assessed using a basic checklist. Therefore, specific details such as timing, description, impact, or severity of the events were not available within the ABCD dataset. Regarding timing, the only information we have is that all participants experienced PTE before the baseline assessment. These details are crucial to consider in order to better understand the factors contributing to trauma adjustment. Additionally, there was no assessment with the BPM-Y before the PTEs occurred, preventing us from comparing problem levels before and after the events. Therefore, it is difficult to determine the extent to which identified trajectories reflect trauma adjustment, or if they are influenced by dealing with other factors, such as adaptation to changes in the course of emerging adolescence or environmental stressors. To differentiate participants by their general frequency of physical activity, we used a composite measure of aerobic and muscle-strengthening activities. However, a limitation of this approach is that it merges two distinct activity types. Future research should consider these categories separately, as they may have different health impacts. Internal consistency (Cronbach’s alpha [α]) for the study variables ranged from excellent to poor (see Table 1). While derived from established instruments, certain scales—particularly those with only three items—yielded lower coefficients. Notably, the Parental Monitoring Scale (PMS) demonstrated low reliability (*α* = 0.45), contrasting with higher consistency reported in previous research [37]. This suggests the PMS items may not have captured a unitary construct within this specific sample. Despite these lower values, all variables were retained to maintain the theoretical integrity of the conceptual model, as they represent core constructs of the study. Excluding them post-hoc based on statistical thresholds would have compromised the theoretical framework. Nevertheless, findings involving these variables should be interpreted with caution, as measurement error may have attenuated the observed associations. The ABCD study sample represents U.S. youth. Nevertheless, caution should be exercised when generalising the results, given the cultural, socioeconomic, and racial/ethnic diversity among different populations. Due to a high proportion of missing data on the BPM teacher report forms, which were only completed once a year, only the self-report form was included. However, triangulating with proxy reports would enhance validity, particularly for externalizing problems. The EM algorithm utilized to compute missing data for determining trajectories is capable of handling complex relationships, preserving data characteristics, being flexible in dealing with different types of data and distributions, robust compared to simpler methods like mean imputation, iterative and readily available in many commercial software packages [66]. However, EM does not fully consider the uncertainty of the imputation, which can result in underestimated standard errors. In contrast to EM, multiple imputation (MI) takes into account uncertainty from missing data by pooling results, but it is computationally more intensive, especially when dealing with large datasets and many imputations [66]. However, in the context of LGMM, the EM algorithm is generally considered the superior approach for handling missing data compared to MI. The EM algorithm provides direct Maximum Likelihood Estimates of the population parameters in a single, efficient process, using all available information for every parameter estimate. The EM algorithm is specifically designed to handle both standard missing data points and unobserved latent classes simultaneously and consistently within the same statistical model. On the contrary, MI can struggle to capture this complex latent structure accurately. MI assumes a single normal distribution, pulling the missing values toward the overall mean, which can introduce bias in handling data containing multiple latent classes. Finally, handling complex data with MI can be challenging due to its stochastic nature [57, 67]. Stekhoven and Buhlmann [68] introduced missForest, which outperformed some of the best and most widely used imputation algorithms available. In terms of imputation accuracy, it is a promising alternative that should be considered in future studies. Although imputation was performed to increase the power of MCAR data for LGMM analysis, it is important to note that imputation was carried out assuming a homogeneous population, whereas LGMM assumes a heterogeneous sample population. Furthermore, the determination of the number of trajectories was not adequately supported by fit indices. Final model selections were based on the BLRT, theoretical justification and interpretability of the results, as recommended by leading researchers in this field [50]. While this ensures conceptual consistency, it must be acknowledged that model selection based on alignment with prior research could also be a source of selection bias. This approach potentially favours models that confirm established findings. Future studies should employ blind model selection or cross-validation techniques to further minimize this risk. It is important to note that the trajectories identified in this study represent latent groups identified through a data-driven research technique. Therefore, group assignment was determined based on probabilities rather than absolute classification criteria. The SHAP results need to be interpreted cautiously, as they represent predictive contributions rather than causal relationships.

## Conclusions and implications

Given the high frequency of PTEs and their potential long-term consequences, it is crucial to implement regular trauma screening into clinical practice when working with children/adolescents. In order to improve mental health care for youth, it is essential to develop accurate and specific clinical diagnostic prediction models for disorders, such as PTSD, depression, anxiety, and ADHD, which are commonly associated with a history of PTEs [20]. When a history of trauma is identified, it is vital to consider both individual (e.g., psychological characteristics and screentime) and social environmental factors (e.g., family conflicts) to predict the course of psychological adjustment. Our findings emphasize the significance of monitoring screen media usage in individual risk estimation and considering it in treatment. Finally, addressing parental symptoms following a child´s PTE is crucial to empower parents to actively participate in their child´s trauma recovery. Since no cut-off and risk score calculations have been conducted to determine whether specific contributing factors represent risks or protective factors, future research needs to establish and validate these criteria. Previous research has indicated that psychological, environmental, individual, and social characteristics are the most commonly identified predictors of outcome trajectories following potential trauma [6]. It is likely that some of the associations between symptom trajectories and predictive features may be stronger in a PTE-exposed sample compared to a community sample where not everyone reports PTEs. Therefore, future research should investigate how the identified predictors impact mental health trajectories within non-exposed youth. Since studies investigating community samples have identified a strong relationship between biological factors (e.g., multitrait polygenic risk scores) and the trajectory of internalizing problems, future studies should investigate the impact of biological predictors on outcome trajectories following potential trauma [69].

## Supplementary Information

Below is the link to the electronic supplementary material.

Supplementary file1 (DOCX 34 KB)

Supplementary file2 (TXT 5 KB)

Supplementary file3 (TXT 79 KB)

## Data Availability

One goal of the ABCD Study is to create a unique data resource for the entire scientific community by embracing an open science model. The ABCD Study releases curated, anonymized data annually and makes it available to the research community. Information on how to access ABCD data is available at: https://abcdstudy.org/scientists/data-sharing/.
