# Supplementary material for: Predicting mental health trajectories after potentially traumatic events: a machine learning approach
Source: Eur Child Adolesc Psychiatry. 2026 Apr 1;35(7):2265–81. doi: 10.1007/s00787-026-03022-6 (PMC13427919; doi:10.1007/s00787-026-03022-6)
Supplement: Supplementary file 1 — Supplementary file1 (DOCX 34 KB) [file 787_2026_3022_MOESM1_ESM.docx]

**Supplementary Table 1**

Description of the entire Adolescent Brain Cognitive Development (ABCD) cohort

| Construct | M (SD)/n (%) |
| --- | --- |
| Child´s age | 9.48 (0.51) |
| Child´s gender: male/female | 6188 (52.1)/5677 (47.8) |
| Child´s race: White/Non-White | 8797 (74.1)/3048 (25.7) |
| Child´s ethnicity: Hispanic/Non-Hispanic | 2410 (20.3)/ 9305 (78.4) |
| Caregiver´s education | 16.59 (2.77) |
| Caregiver´s employment: employed/non-employed | 8210 (69.2)/3602 (30.3) |
| Family income (past year) | 7.22 (2.42) |
| Average screentime (workday: hours) | 3.28 (2.90) |
| BIS (anticipation of punishment) | 9.51 (3.75) |
| BAS: Drive (intensity of goal directed behaviour) | 4.14 (3.06) |
| BAS: Fun Seeking (willingness to approach a potentially rewarding event) | 5.71 (2.65) |
| BAS: Reward Responsiveness (anticipation of reward) | 11.00 (2.92) |
| Prosocial behaviour | 1.68 (0.37) |
| Physical activity | 2.71 (1.74) |
| Parental monitoring | 4.38 (0.52) |
| Struggling expenses: food | 952 (8.0) |
| Evicted from home | 168 (1.4) |
| Struggling expenses: medical | 1358 (11.4) |
| Struggling expenses: other | 1728 (14.5) |
| Parenting/acceptance | 2.78 (0.29) |
| Bullying | 1813 (15.3) |
| Caregiver: internalizing problems | 48.13 (10.55) |
| Caregiver: externalizing problems | 45.95 (9.62) |
| Parents: alcohol/drug problems | 2115 (17.7) |
| Family conflicts | 2.05 (1.95) |
| School: environment | 19.93 (2.83) |
| School: involvement | 13.06 (2.37) |
| School: disengagement | 3.74 (1.46) |
| Neighbourhood | 4.03 (1.10) |
| Internalizing problems | 1.82 (2.09) |
| Externalizing problems | 1.95 (2.00) |

*Note*. Data for all measures, except for youth internalizing and externalizing problems are reported at baseline. Youth´s internalizing and externalizing problems are reported at the 6-month follow-up assessment; Caregiver´s education ranges from 1 (the first grade completed at school) to 21 (Doctoral degree); Family income ranges from 1 (<$5,000) to 10 (≥$200,000); BIS = the Behavioural Inhibition System; BAS = the Behavioural Approach System.

**Supplementary Table 2**

Fit indices, entropies, and percentage of class members for latent growth mixture models

| Internalizing problems | | | | | | |
| --- | --- | --- | --- | --- | --- | --- |
| Number of classes | AIC | BIC | SABIC | LMR-LRT, p-value | Entropy | Percentage: class1/2/3/4/5 |
| 1 | 100950.13 | 100994.43 | 100972.18 |  | 1.000 | 100.0 |
| 2 | 98883.74 | 98959.68 | 98921.55 | < .001 | .64 | 61.0/39.0 |
| 3 | 98254.36 | 98361.95 | 98307.93 | < .001 | .70 | 58.6/8.2/33.2 |
| 4 | 97720.61 | 97859.84 | 97789.94 | < .001 | .69 | 30.8/8.6/48.0/12.5 |
| 5 | 97731.14 | 97902.01 | 97816.22 | 1.000 | .55 | 33.3/44.9/8.4/0/13.4 |
| Externalizing problems | | | | | | |
| 1 | 95234.49 | 95278.79 | 95256.54 |  | 1.000 | 100.0 |
| 2 | 93982.31 | 94058.26 | 94020.13 | < .001 | .53 | 28.3/71.7 |
| 3 | 93578.29 | 93685.88 | 93631.86 | < .001 | .61 | 16.1/34.7/49.3 |
| 4 | 93188.86 | 93328.09 | 93258.19 | < .001 | .64 | 10.4/46.0/34.2/9.4 |
| 5 | 93094.69 | 93265.56 | 93179.77 | < .001 | .61 | 28.5/7.1/23.1/35.0/6.4 |

*Note.* AIC = the Akaike Information Criterion; BIC = Bayesian Information Criterion; SABIC = Sample-Size Adjusted BIC; LMR-LRT = Lo-Mendell-Rubin Likelihood Ratio Test.

**Supplementary Text 1**

The machine learning (ML) pipeline started by addressing missing predictor data using multivariate iterative imputation with a Bayesian Ridge estimator. This was done separately for each target after excluding rows with missing outcomes (externalizing: 49.3% missing; internalizing: 33.2%), resulting in two target-specific datasets. Missing outcomes refer to the trajectory class that was not included in ML. To prevent information leakage, fold-wise imputation was performed within a stratified outer cross-validation loop, fitting the imputer only on training folds and applying it exclusively to predictors. Post-imputation, binary predictors were rounded and continuous predictors were standardized via z-scoring. Subsequently, a coarse model screening with 5-fold cross-validation (CV) and hyperparameter tuning compared Random Forest (bagging), eXtreme Gradient Boosting (XGBoost; boosting), Logistic Regression (Generalized Linear Model [GLM]), SVM (Support Vector Machine; max-margin), and Multilayer Perceptron (MLP; neural network). Random Forest and XGBoost performed best, with SVM and Logistic Regression close behind. Accordingly, XGBoost, SVM, Random Forest (internalizing-only), and Logistic Regression (externalizing-only) were advanced to the full nested CV run. In addition, following this exploratory screening, we evaluated post-hoc probability calibration strategies (Platt scaling and isotonic regression) using cross-validated out-of-fold predictions to assess their impact on probability-based metrics and discrimination. While calibration occasionally improved probability-based loss functions, it did not improve macro-F1 and substantially reduced ROC-AUC by producing more conservative probability estimates and was therefore not incorporated into the final nested CV pipeline.

In nested CV, a 10-inner, 10-outer configuration was followed, with all splits preserving class proportions. Sequential Forward Selection (SFS) was used to identify the most informative feature subsets for each model and target. SFS was run once per outer training fold, with a maximum of 30 features selected per target. The final feature sets were determined by fold-wise consensus, retaining predictors selected in at least 20% of outer folds. We implemented extensive model tuning via Bayesian optimization (Optuna, Tree-structured Parzen Estimator [TPE] sampler) using macro-F1 as the direct cross-validated optimization objective. Because our primary performance target is macro-F1 on an imbalanced binary outcome, our focus was on improving discrimination rather than probability calibration per se. Optuna optimized macro-F1 (the primary metric) averaged across inner folds, using TPE sampler for efficient hyperparameter search. The TPE sampler is a Bayesian optimization algorithm that models the conditional probability of hyperparameters given the observed objective function values, leveraging Gaussian mixture models to approximate the search space. This approach balances exploration and exploitation by iteratively refining the probability distributions of promising hyperparameter configurations, thereby converging toward optimal settings with fewer evaluations compared to grid or random search methods. For XGBoost, tuning for internalizing problems explored 250–500 estimators, tree depths of 5–7, learning rates between 0.001 and 0.004, subsampling rates of 0.65–0.85, column subsampling of 0.70–0.90, L1 regularization (α) from 10⁻⁴ to 5×10⁻², and L2 regularization (λ) from 10⁻⁶ to 10⁻³, whereas for externalizing problems the search focused on 400–500 estimators, depths of 6–7, higher learning rates (0.008–0.02), subsampling of 0.72–0.80, column subsampling of 0.65–0.75, L1 regularization from 10⁻³ to 2×10⁻², and stronger L2 regularization (1–10). In parallel, Random Forest models for internalizing problems optimized the number of trees (150–300) and maximum depth (4–7), while Logistic Regression for externalizing problems concentrated on the regularization strength C within a range 0.05–0.2. Complementing these tree-based and linear models, Support Vector Machines were also tuned in a target-specific manner, using an RBF kernel with C values from 0.3–2.0 for internalizing problems and a linear kernel with C values from 0.001–0.02 for externalizing problems. These focused ranges were informed by the initial exploratory run, where broader search spaces were tested and narrowed around the best-performing parameters.

In our sample the two binary outcomes are moderately imbalanced: internalizing problems - ≈ 12% positives, 88% negatives, while for externalizing problems - ≈ 32 % positives, 68 % negatives. Rather than employing resampling (over‑ or under‑sampling), we adopted cost‑sensitive learning by assigning class weights that inversely reflect the class frequencies. This strategy modifies the loss function so that errors on the minority class are penalized more heavily, preserving all original observations and avoiding the over‑fitting that can arise from duplicated minority samples or the information loss caused by discarding majority instances. Consistent with Zhang et al. [1], who showed cost‑sensitive methods to be more stable and overall superior to both oversampling and undersampling when the data set is not extremely small, we applied class weighting across all models. The approach therefore maintains data integrity while improving performance on the imbalanced targets in our study.

[1] Zhang JW, Lu HJ, Chen WT, Lu Y (2011). A comparison study of Cost-Sensitive learning and sampling methods on imbalanced data sets. Advanced Materials Research. 271:1291-1296. https://doi.org/10.4028/www.scientific.net/amr.271-273.1291

**Supplementary Table 3**

Sequential Forward Selection (SFS) features for a model that involves internalizing problems

| Feature | Selection_count | Selection_frequency | Avg_rank_when_selected |
| --- | --- | --- | --- |
| BIS | 10 | 1 | 2,2 |
| Family conflicts | 10 | 1 | 6,6 |
| Child´s gender | 10 | 1 | 10,6 |
| Polyvictimization | 10 | 1 | 11,5 |
| Caregiver: externalizing problems | 9 | 0,9 | 5,777777778 |
| Fun Seeking | 9 | 0,9 | 14,77777778 |
| Parents: alcohol/drug problems | 9 | 0,9 | 15,55555556 |
| Evicted from home | 9 | 0,9 | 16,77777778 |
| Bullying | 8 | 0,8 | 8,625 |
| Parental monitoring | 8 | 0,8 | 12,25 |
| Child´s age | 8 | 0,8 | 13,125 |
| Reward Responsiveness | 8 | 0,8 | 13,375 |
| Caregiver: internalizing problems | 8 | 0,8 | 13,75 |
| Parenting/acceptance | 8 | 0,8 | 14,25 |
| Domestic violence | 8 | 0,8 | 14,875 |
| Prosocial behaviour | 8 | 0,8 | 17 |
| Child´s race | 8 | 0,8 | 20 |
| School: disengagement | 7 | 0,7 | 9 |
| Drive | 7 | 0,7 | 11,85714286 |
| Child´s ethnicity | 7 | 0,7 | 16,85714286 |
| School: environment | 7 | 0,7 | 17,57142857 |
| Physical abuse | 7 | 0,7 | 17,85714286 |
| Terrorism/war/community violence | 7 | 0,7 | 18,14285714 |
| Other expenses | 7 | 0,7 | 18,28571429 |
| Family income | 6 | 0,6 | 9 |
| School: involvement | 6 | 0,6 | 15 |
| Neighbourhood safety | 6 | 0,6 | 16,66666667 |
| Accidents/natural disasters/fire | 6 | 0,6 | 18 |
| Physical activity | 5 | 0,5 | 10,4 |
| Medical expenses | 5 | 0,5 | 12 |
| PTEs (number) | 5 | 0,5 | 15 |
| Food expenses | 5 | 0,5 | 21,4 |
| Caregiver´s employment | 4 | 0,4 | 13,75 |
| Traumatic loss | 4 | 0,4 | 17,5 |
| Sexual abuse | 4 | 0,4 | 18,25 |
| Caregiver´s education | 2 | 0,2 | 21 |
| Screentime | 2 | 0,2 | 23 |

*Note*. BIS = the Behavioural Inhibition System; PTEs = Potentially Traumatic Events.

**Supplementary Table 4**

Sequential Forward Selection (SFS) features for a model that involves externalizing problems

| Feature | Selection_count | Selection_frequency | Avg_rank_when_selected |
| --- | --- | --- | --- |
| Family conflicts | 10 | 1 | 1 |
| Screentime | 10 | 1 | 2,3 |
| BIS | 10 | 1 | 7,2 |
| Evicted from home | 10 | 1 | 10 |
| Physical abuse | 10 | 1 | 11,6 |
| Terrorism/war/community violence | 10 | 1 | 11,9 |
| Caregiver´s employment | 10 | 1 | 14,8 |
| Sexual abuse | 10 | 1 | 15 |
| School: involvement | 9 | 0,9 | 4,777777778 |
| Caregiver: externalizing problems | 9 | 0,9 | 5,333333333 |
| Other expenses | 9 | 0,9 | 12,88888889 |
| Child´s race | 9 | 0,9 | 13,22222222 |
| Parenting/acceptance | 9 | 0,9 | 14,66666667 |
| Child´s ethnicity | 9 | 0,9 | 16,88888889 |
| Medical expenses | 9 | 0,9 | 17,44444444 |
| Drive | 9 | 0,9 | 17,55555556 |
| Reward Responsiveness | 9 | 0,9 | 19,55555556 |
| Accidents/natural disasters/fire | 8 | 0,8 | 16,125 |
| Child´s gender | 8 | 0,8 | 17,125 |
| Parents: alcohol/drug problems | 8 | 0,8 | 20 |
| Fun Seeking | 8 | 0,8 | 21,875 |
| Traumatic loss | 7 | 0,7 | 17,42857143 |
| Domestic violence | 7 | 0,7 | 18,14285714 |
| Prosocial behaviour | 6 | 0,6 | 15,66666667 |
| PTEs (number) | 6 | 0,6 | 18,5 |
| Food expenses | 6 | 0,6 | 18,5 |
| Polyvictimization | 6 | 0,6 | 19,83333333 |
| Bullying | 6 | 0,6 | 20,66666667 |
| Child´s age | 5 | 0,5 | 13,2 |
| School: disengagement | 5 | 0,5 | 16 |
| School: environment | 5 | 0,5 | 23,4 |
| Physical activity | 4 | 0,4 | 12 |
| Parental monitoring | 4 | 0,4 | 21,75 |
| Caregiver: internalizing problems | 3 | 0,3 | 4,333333333 |
| Neighbourhood safety | 2 | 0,2 | 18,5 |
| Family income | 1 | 0,1 | 17 |
| Caregiver´s education | 1 | 0,1 | 23 |

*Note*. BIS = the Behavioural Inhibition System; PTEs = Potentially Traumatic Events.
